# The Application of Corporate Political Activity Taxonomies to Explore the Lobbying of Ultra-Processed Sugary Food and Drink Industries in Chile: Comment on "Corporate Political Activity: Taxonomies and Model of Corporate Influence on Public Policy"

**DOI:** 10.34172/ijhpm.9154

**Published:** 2025-08-19

**Authors:** Yanela Aravena-Rivas

**Affiliations:** Department of Epidemiology and Public Health, University College London, London, UK.

**Keywords:** Corporate Political Activity, Commercial Determinants of Health, Unhealthy Commodity Industries, Ultra-processed Food, Lobbying

## Abstract

Unhealthy commodities industry actors use different practices, including political practices, to influence policy-making in industry-friendly ways that may result in increased ill health and health inequalities. Ulucanlar and colleagues formulated a comprehensive evidenced-based model and taxonomies to study the corporate political activities of unhealthy commodity industries. This commentary reflects on the process of applying these taxonomies to the study of lobbying of ultra-processed sugary food and drink industries in Chile, a country from the global south that faced strong opposition during the discussion and implementation of legislation to create healthier food environments. The taxonomies were a useful tool to identify and classify the different claims and actions used by ultra-processed sugary food and drink industries when lobbying Chilean authorities. However, there were some challenges in their use that need to be considered when using these taxonomies in similar settings.

 The practices used by commercial actors in pursuit of their economic goals have an important role in shaping the conditions in which people are born, grow, work, live, and age, known as the social determinants of health.^1^ The commercial determinants of health framework by Gilmore and colleagues depicts how commercial sector practices in the areas of finance, politics, science, employment, marketing, supply chains, and reputational management influence social determinants of health, particularly those at the macrolevel such as the political and economic systems. This influence has created a context that enables, rather than regulates, the uncontrolled use of these industry practices, leading to unhealthy and unequal societies.^2^

 Among the diverse set of strategies used by commercial actors to influence public policy, political practices are an essential tool. The influence of the tobacco, alcohol and food industries on the proposal, discussion and passing of public health policies has been documented globally, always with a negative impact on public health policies that aimed to create healthier environments by regulating the access and price to these unhealthy products.^2,3^ Even though similarities in the political practices used by commercial actors have been identified,^4^ these are also context-specific, tailored to the characteristics of the social, political and economic systems in place. For example, political practices in low- and middle-income countries tend to be bolder when compared to high-income countries.^5,6^ Because of this, the study of political practices used by commercial actors in different context-specific scenarios is fundamental to advance upstream public policies aiming to create healthier living conditions. The Corporate Political Activity (CPA) model and taxonomies proposed by Ulucanlar and colleagues aim to provide a comprehensive and flexible evidence-based tool to systematically document the objectives, framing and action strategies used by different commercial actors.^7^ This commentary reflects on the process of applying these taxonomies to describe the lobbying practices of ultra-processed food and drink industries in Chile, particularly regarding the benefits and the challenges of using the new taxonomies proposed by Ulucanlar and colleagues in this specific scenario. The full original research article can be read elsewhere.^8^ However, a short summary is also presented here.

 In an effort to tackle obesity and related health conditions, Chile has implemented a range of upstream public health policies including front-of-package warning labels, advertising restrictions, school-based restrictions, and sugar-sweetened beverages taxation.^9^ All these policies have faced opposition from commercial actors.^10^ At the same time, the country has implemented a Lobby Registry in an effort to improve the transparency and accountability of political actors. Using the information about official meetings between ultra-processed sugary food and drink industries and government officials provided in the Lobby Registry, we identified the main objectives, framing and action strategies used by these actors using a qualitative deductive-inductive approach. We identified 237 records, where the Ministries of Health, Social Development, and Economy were the most frequently lobbied.^8^ Figure summarises the framing and action strategies identified from the most to least frequent.

**Figure:**
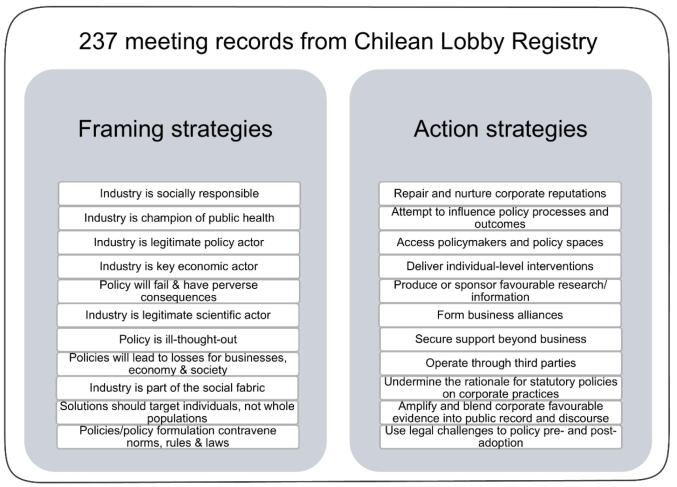
Framing and Action Strategies Used by Ultra-Processed Food and Drink Industries in Chile.

 From the experience of applying the taxonomies in the study previously described, some conclusions can be made that can be of interest to colleagues considering using them in their own research. First, even though the taxonomies were originally created to document a broad range of CPAs with a global perspective in mind, they served well in our study to explore one specific CPA (ie, lobbying) in a specific context (ie, Chile).

 Second, we observed the use of new variations of the taxonomy categories particularly for framing strategies during the lobbying meetings analysed. For example, when industries claimed to be “champions of public health” within the “good actor” category, they not only shared their community responsibility strategies with government authorities but also invited them to join efforts. We also proposed a new claim category for the framing strategies and a new long-term objective for those meetings that could not be directly classified within the existing categories. This happened in part due to the diverse arguments food and drinks industries used when lobbying and to the specific Chilean political and social context. The dichotomy of “good” and “bad” and the active language used to create the taxonomy categories, was sometimes partially masked in the lobby records due to the often friendlier and more subtle wording used by lobbyists when meeting with authorities in these political spaces. However, the bolder nature of CPA was evident in the objectives pursued by the ultra-processed food and drinks industries (eg, requesting changes to public policies or asking to be involved in political decision spaces) and the targeting of high-ranked authorities, including ministers and undersecretaries (ie, the highest ranked authorities within the executive branch of government).

 The CPA taxonomies were created with the intention of minimising the potential overlap between categories, though it is recognised that the composite nature of CPA and their synergistic effects make some overlap inevitable.^7^ In our study, such overlap was commonly observed as several lobbying meetings used a combination of framing strategies. For example, criticising public health policies as “bad” solutions and presenting themselves as “good” actors offering their collaboration to authorities. This should be expected in lobbying activities where there is a need to get the most out of these meetings within the limited time allocated. We also observed that many of the lobbying meetings identified had a synergistic effect with other CPAs such as corporate responsibility activities, scientific, and marketing strategies used by these industries.

 Ulucanlar and colleagues stated that the taxonomies were created with the intention to document, predict, and effectively counter corporate strategies.^7^ We successfully used them to meet the first aim. We documented the diverse set of claims and actions used by ultra-processed food and drinks industries and identified their short- and long-term objectives. This documentation certainly helps to predict future actions and claims, particularly because these industries often use similar strategies.^4^ However, as useful as these taxonomies are to meet the first two aims, they are not sufficient to effectively counter corporate strategies, as this requires specific tools and mechanisms to reduce their influence and manage their power. These taxonomies can however be the first step to developing such tools, which need to be underpinned by a comprehensive understanding of CPAs in different settings.

 The CPA taxonomies are a valuable addition to the study of commercial determinants of health. It is a flexible tool that allows to classify and understand in more depth the corporate political activities of commercial actors. We used it in a specific context to explore one type of CPA and allowed us to comprehensively describe the claims and actions of ultra-processed food and drinks industries in lobbying meetings. The addition of short- and long-term objectives was also helpful to make sense of these strategies in their broader context. CPAs such as lobbying are deeply embedded in the economic, political, social, and scientific systems that shape health and social inequalities such as economic, political, social, and scientific systems. Continuing research on CPAs in different contexts, particularly those targeted by unhealthy commodities industries in recent years such as Latin America is essential to expose and counter strategies leading to unhealthy and unequal systems that have a negative impact on individuals and societies.

## Ethical issues

 Not applicable.

## Conflicts of interest

 Author declares that she has no conflicts of interest.
